# Subconscious Restructuring Protocol in a Mental Health Quality Improvement Study: Reduced Treatment Duration and Improvement Across 17 Psychometric Outcomes in Adult Civilians, Veterans, and Adolescents

**DOI:** 10.7759/cureus.114583

**Published:** 2026-08-15

**Authors:** Kelly Burris

**Affiliations:** 1 Behavioral Health, Burris Institute, Los Angeles, USA

**Keywords:** behavioral microbiome, gut-brain axis, mental health service delivery, pdsa cycles, psychophysiological intervention, quality improvement, routine outcome monitoring, standardized mean change, treatment duration reduction, veteran mental health

## Abstract

Background: Conventional psychiatric and psychotherapeutic services typically require extended treatment durations while collecting limited outcome data, producing inconsistent care. Subconscious Restructuring (SR) uses a behavioral microbiome model treating psychological symptoms and gut barrier integrity as a single clinical target, on the premise that a compromised gut barrier permits translocation of lipopolysaccharide and *Candida*, activating microglia and the neuroinflammation implicated in depression, anxiety, and post-traumatic stress disorder (PTSD). The model is the rationale for the protocol's dual target, not a hypothesis tested here. SR is cloud-based, scripted, and auditable, capturing session-level client-generated data, and has been in clinical use since 1990; it is mechanistic rather than symptom-based, so no personal history is required for delivery. This quality improvement (QI) initiative aimed to improve the efficiency and clinical outcomes of care by delivering a standardized brief emotional fitness protocol through the SR framework.

Methodology: This initiative used Plan-Do-Study-Act cycles with 50 clients across three populations (20 adult civilians, 20 military veterans, 10 adolescents) in multi-site clinical practices. The protocol comprised two two-hour sessions on consecutive days plus three one-hour follow-ups for adult civilians, two two-hour sessions for veterans, and a single four-hour session for adolescents, delivered as extended workshop-format blocks rather than 50-minute appointments. Sixteen psychometric metrics and a derived Stress Load Index were measured, alongside adherence, completion, and safety. The Gut Health Symptom Checklist was completed at every session as a physician-referral gate, not an outcome measure; no gut health data were analyzed. Change was summarized as the mean change in scale points with SD, 95% CI, percent improvement, and within-subject standardized mean change (d_z); paired t-tests are descriptive.

Results: Treatment duration was reduced by 67.5% relative to the 16-hour midpoint of the conventional 12-20-session benchmark, ranging from 56.7% to 74.0% against its 12-hour and 20-hour bounds. Adherence and assessment completion were 100% with no adverse events. Mean improvement across the 17 outcomes was 35.2% for adults, 47.3% for veterans, and 19.1% for adolescents; pooled across 50 clients, depression (Emotional Checklist total) improved 46.63%. Veterans achieved the strongest gains (depression: 57.87%, anger: 63.95%, Stress Load Index: 46.25%). Every outcome improved in raw scale points in all three cohorts, but the adolescent response was smaller and more variable, with 10 of 16 intervals, including zero; its two negative percentages are floor and ceiling artifacts on hopelessness and relationship satisfaction. Standardized Cronbach's alpha was 0.93 for the valence-aligned overall scale, and d_z ranged from 0.04 to 2.04. Reliable improvement on the primary symptom instrument reached 90.0% of veterans, 60.0% of adults, and 50.0% of adolescents; on at least one instrument, these were 95.0%, 95.0%, and 50.0%, respectively.

Conclusions: Standardized brief emotional fitness protocols were associated with improvements in both clinical outcomes and service delivery efficiency. This model offers a replicable framework for reducing treatment timelines; controlled studies are needed to confirm effects on mental health outcomes.

## Introduction

Healthcare systems are facing increasing pressure to deliver efficient, evidence-based mental healthcare while managing resource constraints and addressing access barriers [1]. Traditional mental health treatment typically requires 12-20 sessions over several months [2-4], creating significant challenges for service delivery optimization. This benchmark is consistent with the psychotherapy dose-response literature, which describes a negatively accelerating relationship between the number of sessions delivered and the probability of improvement [2], and which has examined how that relationship translates into the number of sessions associated with clinically significant change in service settings [3]. A systematic review of routinely delivered psychological therapies reported optimal doses ranging from approximately 4 to 26 sessions, depending on setting, clinical population, and outcome measure, and found that weekly scheduling accelerated the rate of improvement relative to less frequent schedules [4]. The 12-20-session range used as the comparison benchmark in this report falls within the span described across that literature, and is used here as a description of typical delivered treatment length rather than as an established requirement. This quality improvement (QI) initiative addresses these challenges with standardized protocols that integrate psychological and physiological assessment approaches along with mechanisms to correct them.

The gut-brain axis represents a bidirectional communication network with established connections to mental health outcomes through neurological, endocrine, and immune pathways [5]. Research indicates that gut microbiota are associated with mood, anxiety, and cognitive function [6,7]. Approximately 500 million neurons line the gastrointestinal tract [8], and the large majority of the body's serotonin, commonly estimated at 90%, is produced there [9,10]. Preclinical work has shown that ingestion of a *Lactobacillus *strain alters emotional behavior and central gamma-aminobutyric acid (GABA) receptor expression in a mouse model via the vagus nerve [11]. The vagus nerve is a principal afferent route within this system, carrying signals from the gut to the brainstem and functioning as an interface between the intestinal microbiota and central regulation of affect [12]; its efferent stimulation has been investigated as a treatment modality in depression [13]. However, systematic integration of gut health measurement into routine mental health practice remains limited. Recent comprehensive reviews further document the role of gut microbiota in mood disorders [14] and the influence of dietary components on anxiety and depression [15].

A specific physiological pathway provides the mechanistic rationale for treating the emotional state and gut health as a single clinical target, which we refer to here as the behavioral microbiome [14,15]. When the intestinal barrier is compromised by microbiome disruption, lipopolysaccharide (LPS) from Gram-negative bacteria can translocate into the systemic circulation, cross the blood-brain barrier, and activate microglia [16,17]. Microglial activation produces neuroinflammation that has been mechanistically linked to major depressive disorder, generalized anxiety, and post-traumatic stress disorder (PTSD) [17]. Under this model, sustained improvement in emotional symptoms is unlikely while a physical breach in the gut barrier permits continuous inflammatory input to the brain.

Human evidence connects this barrier mechanism to affective symptoms. Plasma zonulin and fatty acid binding protein 2, biomarkers of intestinal barrier permeability, have been reported to correlate with plasma LPS and with altered gut microbiome composition in anxiety and depression [18], and a systematic review and meta-analysis has described zonulin-dependent intestinal permeability in children diagnosed with mental disorders [19]. Related work has examined the gut-brain axis in PTSD, describing microbiome alterations alongside the neuroimmune and neuroendocrine pathways proposed to mediate them [20].

The second organism named in this model, *Candida albicans*, is implicated through a distinct route. Colonization with *C. albicans *has been shown in a mouse model to perturb the gut-brain axis through dysregulation of endocannabinoid signaling [21], and *Candida* exposure has been associated with cognitive deficits in schizophrenia and bipolar disorder, with the association differing by sex [22]. The agglutinin-like sequence adhesins of *C. albicans *carry conserved amyloid-forming sequences [23], and amyloid formed by these proteins has been detected in infected human tissue [24]. Microbial functional amyloid is not inert with respect to the host nervous system: exposure to the bacterial amyloid protein curli has been shown to enhance alpha-synuclein aggregation in aged rats and in *Caenorhabditis elegans *[25]. Detection remains a practical constraint on clinical application, as non-culture diagnostics for invasive candidiasis carry recognized limitations alongside their promise [26].

The distinction between what is established and what is proposed should be stated plainly. The proposition that gut barrier integrity and emotional state are usefully treated as a single clinical target is this paper's framing, and it is the rationale for the protocol's design rather than a hypothesis tested by this initiative. The pathway and the point at which the protocol is intended to act are shown schematically in this work. A more complete mechanistic treatment of this pathway is outside the scope of this QI report and is the subject of a separate forthcoming manuscript.

Two terms, conscious and subconscious, are used throughout this paper. The conscious mind refers to deliberate, effortful processing that a person is aware of and can report in words. The subconscious refers to the automatic, rapid, associative processing that stores and runs learned emotional and behavioral patterns outside of awareness. The two differ chiefly in speed, total data capacity, and accessibility to awareness [27,28]. The subconscious mechanisms used in the Subconscious Restructuring (SR) process cannot be clinically measured directly but are evident in measurable outcomes.

The bandwidth available to deliberate verbal processing is a small fraction of that available to the sensory and associative systems that operate outside awareness. A synthesis of behavioral measurements spanning most of a century places the information throughput of human behavior at approximately 10 bits per second, against sensory systems that gather data at approximately 10^9^ bits per second, a differential of roughly eight orders of magnitude [29]. This differential may help explain why initiating behavior change through conventional talk therapy is difficult, since verbal processing operates within the narrower channel while the patterns being targeted were established outside it and are reinforced by accumulated prior programming [30].

The SR framework targets a four-stage sequence: word, picture, emotion, and behavior. The sequence was derived from four decades of clinical observation and outcome tracking, but each transition within it corresponds to an established neurocognitive finding. The initial transition from word to picture aligns with dual coding theory [31] and neuroimaging findings demonstrating that semantic processing automatically activates visual cortices, translating lexical input into mental imagery [32]. The move from picture to emotion draws on evidence that mental imagery drives affective response, often recruiting the same neural activation as direct experience [33].

The link between emotion and behavior rests on appraisal theory, in which appraisal of an event generates a state of action readiness that takes precedence over competing behavior [34]. Separating the stages makes the sequence addressable. Because imagery shapes emotional state, intervening at the word or picture level interrupts the resulting emotional and behavioral output. Bioinformational theory supports this: altering the stimulus representation within an emotional memory network reprograms the response [35]. These subconscious mechanisms do not operate independently of the physiological state, and gut-brain signaling has been shown to influence the affective and cognitive processes on which they depend [5,36].

Identified performance gaps motivating this initiative included average treatment duration of 12-20 sessions, creating access barriers [1-4]; inconsistent outcome measurement across clinical staff, making progress assessment subjective [37]; limited systematic assessment of physiological contributors to psychological symptoms [38], despite established gut-brain connections [5,14]; variable treatment approaches across practitioners, leading to treatment drift [39]; and client feedback indicating a desire for more efficient treatment options [40].

Beyond these operational gaps, three research gaps motivate this work: standardized, brief protocols with routine session-level outcome measurement, systematic integration of gut health assessments into mental health practice, and replicable, client-generated outcome data across distinct populations.

Objectives

Consistent with these gaps, the aim of this initiative was to improve mental health service delivery by implementing a standardized brief mental health protocol, delivered through the SR framework, in order to (1) reduce total treatment duration relative to the traditional 12-20-session standard; (2) achieve measurable within-client improvement across 17 reported outcomes; and (3) maintain protocol adherence, assessment completion, and safety at acceptable thresholds. These three objectives are carried forward, in the same order, through the Materials and Methods, Results, and Conclusions sections.

## Materials and methods

QI framework

This was a single-arm, pre-post QI evaluation conducted under the model for improvement, using iterative Plan-Do-Study-Act (PDSA) cycles to systematically test and refine implementation of standardized protocols across diverse clinical populations. The approach has been iteratively developed since the initial development of SR in 1984 and its introduction into psychiatric care in 1990. Multiple PDSA cycles guided implementation: Plan (develop standardized protocol, train practitioners, establish measurement systems); Do (implement with 50 clients with systematic data collection); Study (analyze outcomes, process measures, and implementation challenges); and Act (refine protocols and plan sustainability). A defining feature of this framework is its reliance on session-level, client-generated data: clients complete the psychometric instruments themselves at every session. Instrument entries made in the Burris Connect platform lock on save: once the client submits a completed instrument, neither the client nor the practitioner can alter the record. This applied to the adult civilian and veteran cohorts. The adolescent cohort completed the instruments on paper, and this control did not apply to that cohort.

Setting and participants

The setting included multi-site clinical practices serving adult civilians, military veterans, and adolescents, with remote cloud-based implementation for adults and veterans and in-office delivery for adolescents. Sessions were structured, scripted SR sessions in which a trained practitioner led the client through the protocol steps, delivered via secure cloud-based software for adults and veterans and in-office for adolescents.

Practitioner Training and Certification

All practitioners completed a standardized certification program that has been in place since 2001 and therefore predates all three data collection periods. The program consists of three four-hour training sessions on consecutive days. Day one covers establishing a clinical baseline using the Burris Connect platform for client data management, including administering the Emotional Fitness Checklist and the Gut Health Symptom Checklist. Day two covers the seven-step protocol, relationship-focused content, and follow-up procedures. Day three covers operational implementation and referral pathways to physicians. Training materials are written in script form, with separate variants for adult, military, and adolescent delivery to standardize delivery and minimize practitioner drift. Certification is not granted on attendance alone: candidates must also complete the full protocol with three clients, collect paired before-and-after data, and conduct follow-up sessions before the practitioner designation is awarded. No clinical licensure is required for certification; the credentials of the three practitioners who contributed data to this initiative are listed in the Acknowledgments. No independent fidelity monitoring, adherence rating, or clinical supervision was conducted during the sessions reported here. Protocol consistency rested on the scripted guidelines, the session-level data captured by the platform for the adult civilian and veteran cohorts, and the paper instruments for the adolescent cohort.

Participants comprised 50 clients across three populations. Adult civilians (n = 20) received a total of seven hours across five sessions: two two-hour sessions on consecutive days, followed by three one-hour follow-up sessions scheduled individually by the client, with a mean interval of seven days. Military veterans (n = 20) received a total of four hours across two two-hour sessions on consecutive days. Adolescents (n = 10) received a single four-hour session with modified protocol elements. Follow-up sessions were delivered to the adult civilian cohort only.

Session Format

Contact was delivered as extended workshop-format blocks rather than as a series of consecutive 50-minute outpatient appointments. The two-hour and four-hour blocks are intrinsic to the protocol, which builds each step on the material established in the preceding step within a single sitting, and they are the format in which the protocol has been delivered in routine practice since 1990. Assessment burden within each block is low: the three outcome instruments are administered together as a single 22-item form that clients complete in approximately three minutes, and the Gut Health Symptom Checklist is a separate 22-item form that clients also complete at every session. The stated contact durations therefore refer to the protocol delivery time, not to the time spent completing instruments.

Cohort Selection

These three cohorts were selected to confirm, under systematic measurement, what clinical application of the protocol since 1990 had indicated: that a scripted, mechanism-focused process, which operates without diagnostic labels or personal history exploration, holds up across substantial variation in age, presenting concerns, delivery setting, practitioner, and session structure. A symptom-focused approach requires excavation of personal history and reformulation for each presentation and setting; a mechanism-focused one does not. Adult civilians, military veterans under treatment for service-related trauma, and adolescents differ on every one of those dimensions, and the protocol was delivered from an identical script to all three. Improvement was observed in every measured domain in all three cohorts, within four to seven hours of contact time. The variation did not impede improvement. This is the pattern a common-process account predicts, though a single-arm design without a symptom-focused comparator cannot establish it.

Recruitment

Both the adult civilian and veteran cohorts were assembled using consecutive convenience sampling; every eligible client who enrolled during the recruitment window was included. Adult civilian participants enrolled in SR workshops during the study period. Veteran participants were recruited by the practitioner in his role as Peer Liaison with the Shaping Hope and Recovery Excellence (SHARE) Military Initiative at Shepherd Center in 2011; all had served in the United States armed forces and were undergoing treatment for service-related trauma, traumatic brain injury (TBI), and/or behavioral health conditions, including PTSD, depression, and anxiety. The adolescent cohort was a convenience sample: children of friends and family members associated with the practice were identified, a parent or guardian was given study information and provided signed consent, and the adolescent was then enrolled and assented to participate.

Eligibility

For the adult cohort, no formal eligibility restrictions were applied beyond enrollment in the SR workshop. Veteran participants were United States military service members and veterans receiving care through the SHARE Military Initiative. The adolescent cohort was restricted to ages 13 -18 and required a signed parental release form. Exclusion criteria were inability to complete the self-report instruments (adult and veteran cohorts); for the adolescent cohort, age outside the range of 13-18 years or absence of a signed parental release. Any client scoring 5 or above on the suicidal-ideation item (Emotional Checklist item 12) was referred for higher-level care under the safety protocol. Meeting this threshold was not an exclusion criterion, and no client was excluded from the analyzed sample on this basis. Threshold crossings and their disposition are reported in the Results section.

Sociodemographic

Consistent with the mechanistic, rather than symptomatic, operationalization of this protocol, sociodemographic variables, such as race, education, and employment, were not collected, and clients were not assigned diagnostic labels; however, a diagnosis could be extrapolated from the psychometric data if needed. Age and sex were recorded and are reported in the Results section. The three cohorts were not contemporaneous: veteran data were collected in 2011, adolescent data in 2016, and adult data in 2018.

Ethical considerations

This QI initiative was conducted as part of routine clinical care evaluation. Because the Burris Institute maintains no standing institutional review board (IRB), the principal investigator determined the project to be exempt from review as a QI activity not constituting human subjects research, in accordance with 45 CFR 46.102(l) and US Office for Human Research Protections (OHRP) guidance (determination dated June 5, 2025). All adult and veteran participants agreed that their de-identified data could be used for study and evaluation; for the adolescent cohort, a parent or guardian provided authorization through a signed release form. All data were de-identified prior to analysis. Comprehensive safety protocols were implemented, including immediate referral for clients scoring 5 or above on the suicidal-ideation question.

Standardized SR protocol

A seven-step SR framework targeting subconscious processing mechanisms, with an integrated gut health referral gate, was implemented as follows:

Step 1 (Emotional Fitness and Gut Health Checklists)

The practitioner introduces the measurement framework and explains the gut-brain connection. The client completes the Emotional Checklist (a 12-item self-report measure scored on a 1-10 Likert scale; normal range: 1-4), the Behavior Control Checklist (normal range: 7-10), the Relationship Satisfaction Scale (normal range: 7-10), and the Gut Health Symptom Checklist (normal range: 1-4). The adult civilian and veteran cohorts completed the instruments themselves in the Burris Connect platform; the adolescent cohort, seen in the office, completed them on paper. Both the Emotional Fitness Checklist and the Gut Health Symptom Checklist are completed by the client at every session, not only at intake. Step 1 describes their first administration, and the same pair of instruments is used at each subsequent session. The Emotional Fitness Checklist and Gut Health Symptom Checklist are presented in the Appendix.

Step 2 (Subconscious Perspective and Empowering Questions)

Internal dialogue, or questions, constitutes the first component of the word-picture-emotion-behavior equation. The client responds to four statements and one question, after which the practitioner breaks down how the structure of questions determines whether the client moves toward or away from their objectives. The practitioner then explains how to structure internal questions so that the client consistently moves toward their objectives. This mechanism addresses the initial word stage of the subconscious sequence, directing the mind to generate solutions rather than focusing on limitations.

Step 3 (Subconscious Self-Image)

The practitioner asks the client how their internal pictures are structured, explains the importance of that structure, and provides examples of differences. The client then uses the inner dialogue structure established in Step 2 to form, change, and consistently take control of their internal pictures. This mechanism addresses the picture stage of the word-picture-emotion-behavior sequence, placing the imagery that drives emotion and behavior under the client's deliberate control.

Step 4 (The Stop and Replace System)

The practitioner explains that the Stop and Replace System applies all components from the first three steps to fully restructure a subconscious program, producing an emotional state and behavior that does not work, beginning with the three most limiting emotional states (fear, guilt, and anger), unless the client identifies something else. For each emotion, the client writes the benefits of not engaging in the behavior with a correlating empowering question for each, describes the objective picture (associated) and the old picture (dissociated), and identifies the cue by backing up the old picture to the moment just before the behavior begins.

The practitioner then teaches the switch pattern: upon recognizing the cue, the client says "stop" to destroy the old picture and "replace" to simultaneously bring up the objective picture. The client rereads every Stop and Replace sheet daily until each targeted habit, behavior, or emotion has been changed to their satisfaction. This mechanism enables the client to interrupt, restructure, and reprogram the sequence of words, pictures, emotions, and behaviors in real time.

Step 5 (The Heart of Subconscious Restructuring)

The practitioner introduces the Heart of Subconscious Restructuring, which organizes the client's ongoing internal dialogue across four categories: love, health, wealth, and self-image. The practitioner presents five key questions: three used throughout the day (Does this work for me? How do I feel, and will I benefit from the results of this? What can I replace this with that will benefit me?) and two used in place of self-reprimand (What can I learn from this? How can I use this to move more quickly toward my objectives?). The client asks a minimum of one question per category every morning and every night, adapts or creates questions to suit their needs, and continues restructuring their written empowering questions until each is satisfactorily answered. This mechanism sustains the restructuring process by maintaining daily conscious control over the internal dialogue that initiates the sequence of words, pictures, emotions, and behaviors.

Step 6 (SR Gut Barrier and Microbiome Restoration)

The practitioner reviews the Gut Health Symptom Checklist with the client to educate them on how gut barrier dysfunction and microbiome disruption drive systemic inflammation and emotional dysregulation through the gut-brain axis. Where a client scores 5 or above on any item, the practitioner asks how long that symptom has been present at that level. A client reporting a duration of 30 days or longer is referred to a physician for evaluation under the Test, Do Not Guess principle. The client-facing form carries a general recommendation to consult a functional medicine-qualified healthcare provider for scores of 5 or above; the duration question is the practitioner-applied criterion. The checklist is presented with a disclaimer, which the client must acknowledge before the instrument can be completed, stating that the content is educational, that it is not to be used to diagnose or treat a health problem or disease, that no clinical relationship is created by its use, and that any protocol is to be implemented only under the supervision of a physician, with the SR practitioner in a support role. The client tracks daily food, fitness, and sleep patterns through the Food and Fitness Planner. The client's recovery is organized into a three-phase framework (assessment, targeted action, and stabilization and maintenance) implemented only under medical supervision, with the practitioner limited to a support role. This mechanism addresses the physiological barriers to emotional health, operating on the premise that a compromised gut barrier perpetuates systemic inflammation, neuroinflammation, and mood disruption until the barrier and microbiome are restored.

Step 7 (The Trance-Formation)

The practitioner introduces the Trance-Formation, a light trance state that the client uses to communicate directly with the subconscious and consolidate the material built in the first six steps. Unlike forms of meditation that aim to quiet the mind, the client remains fully aware and in control while working with the words and pictures specific to them, their empowering questions, and their objective pictures, rather than generic content. Guided by the induction script (delivered by a practitioner experienced in guided meditation, or read by the client, or recorded as an audio), the client descends a visualized staircase into a relaxed state, brings up the associated objective picture, and reshapes it until it matches what they want, applying the Stop and Replace System whenever they drift off course. The client enters this light trance at least once daily. This mechanism uses light trance as accelerated restructuring, giving the client deliberate control over the empowering questions and images that drive the word-picture-emotion-behavior sequence, thereby consolidating the new self-image and behavioral pathways.

Maintenance: The client sustains results through daily self-directed practice: rereading the Stop and Replace sheets, converting excuses into empowering questions as they arise, and returning to the Subconscious Perspective when focus on objectives is lost. If a thought, emotion, or behavior that does not work returns, the client asks how the positive change was achieved previously and repeats those actions, rather than treating recurrence as a failure. This mechanism places ongoing control of the word-picture-emotion-behavior sequence in the client's hands, maintaining the restructuring through consistent daily application of the tools built across the seven steps.

Follow-up: The protocol recommends structured follow-up a minimum of once per week for 30 days, with the schedule adapted by cohort. In this initiative, follow-up sessions were delivered to the adult civilian cohort only, at a mean interval of seven days across three sessions; individual session intervals beyond the cohort mean were not retained as study data. The veteran and adolescent cohorts completed their intervention within the initial session structure and received no scheduled follow-up. Every session opens with the Emotional Fitness Checklist and the Gut Health Symptom Checklist to compare current scores against baseline, with the first three items corresponding to the fear, guilt, and anger states worked in the Stop and Replace System. The practitioner reinstalls the subconscious model at each session, initially explaining it and later having the client explain it back. This mechanism monitors whether the restructuring is holding, with the checklist functioning as the objective measure of client progress and the trigger for referral when scores do not resolve.

Measurement framework

Outcome measures used three instruments, which together comprise the Emotional Fitness Checklist: the Emotional Checklist (12 items, 1-10 Likert), the Behavior Control Checklist (five items), and the Relationship Satisfaction Scale (five items). All items on all three instruments are scored on the same 1-10 Likert metric, and no subscales are computed within any instrument. On the Emotional Checklist, a lower number is preferable; on the Behavior Control Checklist and Relationship Satisfaction Scale, a higher number is preferable. Score interpretation bands for the three instruments are evaluated to determine whether a client falls within a normal, healthy range. For the Emotional Checklist (12 items; total possible range: 12-120), the normal range is 1-4 per item. In this study, depressive symptom burden is operationalized as the Emotional Checklist total, on which a per-item score of 1-4 (total 12-48) is treated as the normal range, and higher totals indicate greater burden. This is an operational definition specific to this instrument, not a validated diagnostic construct, and no criterion measure of depression was administered. Two items are compound in wording: item 5 asks about sadness or hopelessness together, and item 9 asks about loss of appetite or compulsive overeating. Both are scored as single items, and the derived metrics, labeled Hopelessness and Eating Behavior, should be read accordingly. For the Behavior Control Checklist (five items; total possible range: 5-50) and the Relationship Satisfaction Scale (five items; total possible range: 5-50), the normal range is a score of 7-10 per item. Administered together, they form the 22-item Emotional Fitness Checklist, with a total possible range of 22-220. No interpretation band is defined for the pooled 22-item total because the constituent instruments are keyed in opposite directions. The pooled scale is used only for the valence-aligned internal-consistency coefficient reported in the Results section, and its interpretation applies at the level of the individual instruments.

The Gut Health Symptom Checklist comprises 22 items across three symptom sections: gallstones and liver stones (seven items), parasites (seven items), and small intestinal bacterial overgrowth (SIBO), *Candida*, irritable bowel syndrome (IBS), and gut dysbiosis (eight items). Each item is scored on the same 1-10 Likert scale, anchored at 1 (not at all) and 10 (a lot), with a section total recorded for each item. The instrument is explicitly non-diagnostic and is accompanied by a disclaimer that the client must acknowledge before completing the instrument, stating the non-diagnostic limitation and directing the respondent to a qualified healthcare provider (Step 6). It functioned as a referral gate rather than an outcome measure, used to confirm whether the client's physiological status supports their emotional-health objectives, under the referral criterion described in Step 6. The checklist is a specified component of the protocol and is completed by the client at every session. Structured capture and retention of the completed responses as a searchable record in the Burris Connect platform did not begin until 2021, which post-dates all three data collection periods. No retained gut health data were therefore available for analysis, and no gut health scores are reported as study outcomes.

Process measures included protocol adherence, completion of psychometric assessments, and implementation of safety protocols, including referrals when indicated. Adverse events were monitored as a balancing measure. Client satisfaction and resource utilization were not systematically collected in this initiative and are therefore not reported. Process, safety, and balancing measures are summarized in the Results section.

The instruments used in this study, the Emotional Fitness Checklist (incorporating the Emotional Checklist, Behavior Control Checklist, and Relationship Satisfaction Scale) and the Gut Health Symptom Checklist, are digital tools integrated into the Burris Connect clinical platform. These instruments are made available at no cost, developed for this framework, and are reproduced in full in the Appendix. No formal pilot study or construct validity testing was conducted; they are original clinical tools derived and iteratively refined over approximately 40 years of continuous clinical practice through structured client feedback. Because this was a single-arm evaluation without an external criterion, internal-consistency reliability and within-subject effect sizes serve as the primary basis for the psychometric reporting.

SR psychometric operationalization

From the Emotional Checklist: depression (total), anxiety (item 1), negative self-talk or guilt (item 2), anger (item 3), sleep (item 4), hopelessness (item 5), self-esteem (item 6), eating behavior (item 9), decision-making (item 10), hatred toward self or others (item 11), and suicidal ideation (item 12).

From the Behavior Control Checklist: love as an emotional state (item 3), self-confidence (item 4), and motivation and focus (item 5).

From the Relationship Satisfaction Scale: communication (item 1) and relationship satisfaction (total). These yield 16 directly measured metrics.

A 17th reported outcome, the Stress Load Index, is the mean of the percent improvements across the three instruments and is reported alongside but not treated as an independent measurement.

Statistical analysis

All analyses were performed on the de-identified, session-level data captured in the secure cloud-based platform. Because this was a single-arm QI evaluation with paired (before-and-after) observations and no comparison group, analyses are descriptive and within-subject.

Primary and Secondary Outcomes

The primary outcome for each metric was the mean change in scale points from before to after, with its SD. Percent improvement is reported as a secondary, descriptive figure, computed per client according to instrument direction. The equations are as follows:

Symptom-reduction items (Emotional Checklist): percent improvement = (Before - After) / Before x 100

Positive-direction items (Behavior Control Checklist, Relationship Satisfaction Scale): percent improvement = (After - Before) / After x 100

Stress Load Index = mean of the three instrument-level percent improvements

Population values are the mean of the per-client ratios and are therefore sensitive to low baseline or follow-up scores, which can produce large or negative percentages where raw point changes are small but positive (a floor effect). Raw point changes are treated as the authoritative summary.

Stress Load Index and Mean Improvement

Stress Load Index and the aggregate mean improvement are computed at different levels and therefore differ. The Stress Load Index is the mean of only three instrument-level percent improvements (the Emotional Checklist, Behavior Control Checklist, and Relationship Satisfaction Scale totals), whereas the aggregate mean improvement is the mean of all 17 reported outcomes, comprising the 16 measured metrics and the Stress Load Index. Because they average over different sets of quantities, the two values are not expected to match.

Confidence Intervals

Ninety-five percent confidence intervals for the mean change on each metric were computed on the raw client-level paired difference scores as the mean change plus or minus t at the 0.975 quantile times the standard error of the change, with degrees of freedom equal to n minus 1. Intervals are uncorrected for multiple comparisons and are descriptive rather than confirmatory. They are reported alongside the mean changes. The interval for the Stress Load Index is computed on the client-level composite and is expressed in percentage points rather than scale points.

Effect Sizes

Within-subject standardized mean change (d_z) was computed as the mean change divided by the standard deviation of the change scores from the raw client-level difference scores.

Reliable Change

To express outcomes on a client-level metric comparable to the psychotherapy outcome literature, the Jacobson-Truax Reliable Change Index (RCI) was computed for each client on each instrument total [41]. The standard error of measurement was derived from the baseline standard deviation of each instrument within each cohort, and that instrument's standardized Cronbach's alpha and the standard error of the difference were calculated as the square root of twice the squared standard error of measurement. A client was classified as showing reliable improvement when the change score in the favorable direction exceeded 1.96 standard errors of the difference. Because no normative or clinical cut-off scores exist for these original instruments, the clinically significant change criterion of Jacobson and Truax, which requires crossing a normative threshold, could not be applied; reliable change is therefore reported alone.

Significance Testing

Paired two-tailed t-tests were compared before and after scores within each population for every metric, alpha 0.05, computed on the raw client-level difference scores. Prior to testing, the distribution of each change-score set was assessed using the Shapiro-Wilk test. Of the 48 distributions (16 metrics across three cohorts), 33 were consistent with normality (p >= 0.05), and 15 showed departure at p < 0.05, most often on items with restricted range or floor effects such as suicidal ideation, eating behavior, and sleep quality. Because a within-subject paired design was used, homogeneity of variance across independent groups was not applicable. Given the departures observed, a sensitivity analysis was conducted in which every comparison was repeated using the Wilcoxon signed-rank test. The nonparametric and parametric results agreed on statistical significance at alpha 0.05 in all 48 comparisons, indicating that the reported t-test results are robust to the distributional departures. Degrees of freedom were n - 1 (df = 19 for adults and veterans, df = 9 for adolescents, df = 8 for the adolescent Love cell, where n = 9). Because 16 metrics were examined per population, these tests were not corrected for multiple comparisons and are reported as descriptive rather than confirmatory.

Gut Health Analysis

No gut health analysis was performed. Physician referrals arising from the Step 6 criterion are reported descriptively.

Missing Data

Missing data were handled by complete-case analysis. One adolescent participant left two Behavior Control Checklist items blank (items 1 and 3), so adolescent love (emotional state) is based on n = 9. That participant's behavior control total was calculated from the three completed items and was unchanged from before to after, so instrument-total analyses retain n = 10, and the participant did not meet the reliable-change criterion under either treatment for the missing entries. The adolescent Stress Load Index is the single exception and is reported at n = 9, because that composite is a per-client average of three instrument-level percent improvements and was restricted to clients with all three instruments complete at the item level. No imputation was performed.

Reliability. Internal consistency (standardized Cronbach's alpha) was computed for each instrument and overall in the study sample at baseline. Formal construct and criterion validity analyses were not undertaken because the single-arm design, sample size, and absence of an external criterion did not support them; internal-consistency reliability and within-subject effect sizes are therefore the basis for the psychometric claims.

All mean changes, SDs, percent improvements, Stress Load Index values, d_z values, paired t-tests, and Cronbach's alpha coefficients were computed from the present 50-client dataset.

## Results

Baseline characteristics are presented first (Table 1), followed by service-delivery findings and then outcomes by population and cross-population patterns.

**Table 1:** Baseline characteristics and study parameters by cohort Categorical variables are reported as n (%) and age as mean (SD) in years. Cohorts were collected in separate years (2011, 2016, 2018) and were not contemporaneous.

Characteristic	Adults	Veterans	Adolescents
Total clients, n	20	20	10
Male, n (%)	4 (20.0)	15 (75.0)	2 (20.0)
Female, n (%)	16 (80.0)	5 (25.0)	8 (80.0)
Mean age, yr (SD)	39.0 (14.0)	31.0 (4.5)	15.9 (1.4)
Year of data collection	2018	2011	2016
Intervention structure	5 sessions/7 h	2 sessions/4 h	1 session/4 h

The adult civilian cohort comprised 20 clients, 4 male with a mean age of 44.0 years and 16 female with a mean age of 37.7 years, giving an overall mean of 39.0 years. The veteran cohort comprised 20 clients, 15 male with a mean age of 30.6 years and 5 female with a mean age of 32.2 years, giving an overall mean of 31.0 years. The adolescent cohort comprised 10 clients, two male with a mean age of 16.0 years and eight female with a mean age of 15.9 years, giving an overall mean of 15.9 years. Overall means are the count-weighted means of the male and female group means. The cohorts therefore differ substantially in both age and sex composition.

Service delivery and safety

Across all 50 clients, protocol adherence and completion of the psychometric assessments were both 100%, and no adverse events were recorded. Total treatment duration decreased from the conventional benchmark of 12-20 sessions (approximately 12-20 hours) to a weighted mean of 5.2 hours of intervention across the 50 clients (adults: seven hours across five sessions; veterans: four hours across two sessions; adolescents: four hours in a single session). Relative to the 16-hour midpoint of the conventional benchmark, this represents a 67.5% reduction in treatment duration (range: 56.7%-74.0% against the 12-hour and 20-hour bounds, respectively).

Basis of the Duration Comparison

The conventional benchmark is expressed in clock hours on the convention that a billed individual psychotherapy session occupies a full hour. The duration reduction reported throughout this paper is therefore expressed on a clock-hour basis, as a range of approximately 56.7%-74.0% relative to the benchmark's 12-hour and 20-hour bounds, with 67.5% as the point estimate relative to the 16-hour midpoint.

Seven of the 50 clients scored 5 or above on the suicidal-ideation item (Emotional Checklist item 12) during the initiative: six military veterans and one adult civilian. All six veterans scored below the threshold following intervention. The adult civilian (client 17) scored 5 following intervention, was offered referral to a psychiatrist under the safety protocol, and declined. No adolescent reached the threshold at any assessment. No physician referrals arising from the Step 6 gut health criterion were recorded during the study period. Process, safety, and balancing measures are summarized in Table 2.

**Table 2:** Process, safety, and balancing measures across all cohorts Process measures are protocol adherence, completion of the psychometric assessments, and safety protocol implementation. Adverse events, client satisfaction, and resource utilization were pre-specified as balancing measures; the latter two were not systematically collected in this initiative, and no values are reported for them. Safety rows summarize crossings of the suicidal-ideation referral threshold and their disposition. The Step 6 referral row reflects physician referrals arising from the gut health criterion, none of which were recorded during the study period.

Measure	Type	Result	Denominator
Protocol adherence	Process	100%	50 of 50 clients
Completion of psychometric assessments	Process	100%	50 of 50 clients
Safety protocol implementation (referral offered when indicated)	Process	100%	7 of 7 threshold crossings
Adverse events	Balancing	0	50 clients
Suicidal-ideation threshold crossings (Emotional Checklist item 12, score >= 5)	Safety	7 (6 veterans, 1 adult civilian)	50 clients
Threshold crossings resolving below the criterion after intervention	Safety	6 of 7	7 crossings
Referral offered and declined after intervention	Safety	1 (adult civilian, client 17)	7 crossings
Gut health physician referrals recorded (Step 6)	Process	None recorded; checklist administered at every session, but responses not retained in structured form before 2021	Not applicable
Client satisfaction	Balancing	Not systematically collected	Not applicable
Resource utilization	Balancing	Not systematically collected	Not applicable

Baseline and post-treatment scores

Baseline and post-treatment means with SDs for all 16 metrics are presented in Table 3. Raw mean changes with SDs and 95% confidence intervals are presented in Table 4.

**Table 3:** Baseline and post-treatment means (SD) by cohort Each cell is the mean with its standard deviation in parentheses, computed from client-level data (adults n = 20, veterans n = 20, adolescents n = 10). Depression and relationship satisfaction are instrument totals; all other rows are single items. Adolescent love (emotional state) is n = 9, one participant having a missing behavior control entry.

Metric	Adults (pre)	Adults (post)	Veterans (pre)	Veterans (post)	Adolescents (pre)	Adolescents (post)
1. Depression	70.85 (23.60)	38.80 (18.21)	78.50 (21.12)	31.30 (16.68)	42.50 (16.90)	28.90 (13.92)
2. Anxiety	8.20 (2.21)	4.45 (2.26)	8.15 (1.90)	3.75 (2.20)	7.10 (3.14)	3.80 (2.15)
3. Negative Self-Talk (Guilt)	6.90 (2.17)	3.60 (2.30)	7.50 (2.40)	3.35 (2.21)	5.00 (2.91)	2.80 (2.25)
4. Anger	6.05 (2.93)	3.70 (2.36)	7.70 (2.41)	2.65 (1.79)	4.10 (2.77)	1.60 (1.26)
5. Sleep Quality	6.60 (3.15)	4.30 (2.77)	8.00 (1.84)	3.35 (2.25)	2.50 (1.65)	1.80 (1.32)
6. Hopelessness	5.65 (3.00)	2.80 (1.77)	6.25 (2.77)	2.05 (1.19)	3.00 (2.67)	2.30 (1.49)
7. Self-Esteem	5.75 (3.02)	2.90 (1.94)	5.40 (3.19)	1.85 (1.23)	2.70 (2.67)	1.80 (1.48)
8. Eating Behavior	5.50 (3.24)	3.05 (2.39)	5.60 (3.00)	2.55 (1.90)	3.50 (2.42)	2.80 (2.44)
9. Decision-Making	6.10 (3.26)	3.60 (1.90)	6.65 (2.11)	2.95 (2.31)	4.50 (3.03)	3.90 (3.11)
10. Hatred (Self-Others)	5.10 (3.32)	2.35 (1.39)	6.25 (3.11)	2.80 (1.77)	2.60 (1.96)	2.40 (2.17)
11. Suicidal Ideation	3.35 (2.66)	1.60 (1.05)	3.40 (2.41)	1.15 (0.49)	1.30 (0.48)	1.00 (0.00)
12. Love (Emotional State)	3.30 (2.36)	6.55 (2.28)	3.75 (2.38)	7.50 (1.85)	4.56 (2.60)	8.89 (1.62)
13. Self-Confidence	4.90 (2.36)	7.60 (1.96)	5.40 (2.28)	8.60 (1.39)	7.40 (3.06)	8.80 (1.87)
14. Motivation and Focus	4.85 (2.43)	7.00 (2.00)	4.85 (2.37)	8.20 (1.58)	6.20 (2.94)	8.50 (2.07)
15. Communication	4.95 (2.09)	7.15 (1.98)	4.85 (2.37)	7.50 (2.01)	7.20 (3.16)	7.70 (2.79)
16. Relationship Satisfaction	29.25 (9.12)	37.25 (8.17)	24.10 (8.09)	39.05 (9.11)	38.30 (9.25)	38.50 (10.86)

**Table 4:** Mean change in scale points (SD) with 95% confidence intervals by population Each cohort is reported as the mean change with its standard deviation in parentheses, followed by the 95% confidence interval. The Scale column gives the instrument range. In raw scale points, every metric improved in all three populations, although 10 of the 16 adolescent intervals include zero. Intervals were computed on the raw client-level paired difference scores as the mean change plus or minus t at the 0.975 quantile times the standard error of the change, with df = n minus 1 (19 adults, 19 veterans, 9 adolescents; 8 for adolescent love (emotional state), where n = 9), and are uncorrected for multiple comparisons.

Metric	Scale	Adults change (SD)	Adults 95% CI	Veterans change (SD)	Veterans 95% CI	Adolescents change (SD)	Adolescents 95% CI
1. Depression	12-120	32.0 (20.0)	22.6 to 41.4	47.2 (26.3)	34.9 to 59.5	13.6 (11.2)	5.6 to 21.6
2. Anxiety	1-10	3.8 (2.5)	2.61 to 4.99	4.4 (2.2)	3.39 to 5.41	3.3 (1.9)	1.91 to 4.69
3. Negative Self-Talk (Guilt)	1-10	3.3 (1.8)	2.48 to 4.12	4.2 (2.8)	2.86 to 5.54	2.2 (2.7)	0.27 to 4.13
4. Anger	1-10	2.4 (3.4)	0.80 to 4.00	5.0 (2.6)	3.78 to 6.22	2.5 (2.2)	0.95 to 4.05
5. Sleep Quality	1-10	2.3 (2.6)	1.08 to 3.52	4.7 (2.9)	3.34 to 6.06	0.7 (1.3)	-0.19 to 1.59
6. Hopelessness	1-10	2.9 (2.2)	1.87 to 3.93	4.2 (2.7)	2.94 to 5.46	0.7 (2.5)	-1.08 to 2.48
7. Self-Esteem	1-10	2.9 (2.7)	1.63 to 4.17	3.5 (3.0)	2.10 to 4.90	0.9 (1.4)	-0.08 to 1.88
8. Eating Behavior	1-10	2.5 (2.8)	1.18 to 3.82	3.0 (3.4)	1.45 to 4.55	0.7 (1.6)	-0.42 to 1.82
9. Decision-Making	1-10	2.5 (2.7)	1.24 to 3.76	3.7 (3.0)	2.31 to 5.09	0.6 (1.7)	-0.62 to 1.82
10. Hatred (Self-Others)	1-10	2.8 (3.2)	1.28 to 4.32	3.5 (3.0)	2.07 to 4.93	0.2 (1.0)	-0.54 to 0.94
11. Suicidal Ideation	1-10	1.8 (2.1)	0.76 to 2.84	2.2 (2.1)	1.22 to 3.18	0.3 (0.5)	-0.05 to 0.65
12. Love (Emotional State)	1-10	3.2 (2.5)	2.05 to 4.35	3.8 (2.9)	2.42 to 5.18	4.3 (3.2)	1.89 to 6.71
13. Self-Confidence	1-10	2.7 (1.9)	1.83 to 3.57	3.2 (2.6)	2.00 to 4.40	1.4 (2.2)	-0.19 to 2.99
14. Motivation and Focus	1-10	2.1 (1.8)	1.27 to 2.93	3.4 (2.2)	2.35 to 4.45	2.3 (1.9)	0.91 to 3.69
15. Communication	1-10	2.2 (2.2)	1.16 to 3.24	2.6 (3.1)	1.18 to 4.02	0.5 (2.1)	-1.01 to 2.01
16. Relationship Satisfaction	5-50	8.0 (8.3)	4.1 to 11.9	14.9 (12.0)	9.3 to 20.5	0.2 (5.2)	-3.6 to 4.0

Outcomes by population

Adult Civilian Population (n = 20)

The adult civilian population improved across all 17 psychometric outcomes following seven hours of intervention. The most notable improvements occurred in primary symptoms: negative self-talk or guilt at 50.64% (the greatest individual improvement), love as an emotional state at 48.02%, anxiety at 44.48%, depression at 44.03%, hopelessness at 43.69%, and self-esteem at 40.82%. Behavioral and cognitive metrics included self-confidence at 36.87%, eating behavior at 35.61%, sleep quality at 31.54%, motivation and focus at 31.51%, and suicidal ideation at 31.45%. Interpersonal and emotional-regulation metrics included decision-making at 28.00%, communication at 27.94%, anger at 24.30%, hatred toward self or others at 22.79%, and relationship satisfaction at 20.58%. The Stress Load Index improved to 36.49% +/- 14.78.

Veteran Population (n = 20)

Veterans improved across all metrics. Highest-performing metrics were anger at 63.95% (the highest across all populations), hopelessness at 58.93%, depression at 57.87%, sleep quality at 55.79%, anxiety at 54.47%, negative self-talk at 51.37%, and self-esteem at 50.68%. Moderate-to-strong improvements included love at 47.68%, suicidal ideation at 46.29%, hatred at 44.32%, decision-making at 43.72%, motivation and focus at 41.16%, and eating behavior at 39.43%. Interpersonal functioning showed self-confidence at 35.93%, relationship satisfaction at 34.99%, and communication at 30.47%. The Stress Load Index improved 46.25% +/- 18.86, the highest across all populations.

Adolescent Population (n = 10)

Adolescents showed variable outcomes following a single four-hour intervention. Strong positive responses included anger at 49.19% (the highest for this population), love at 45.83%, anxiety at 42.90%, negative self-talk at 34.28%, motivation and focus at 29.37%, and depression at 29.33%. Moderate improvements were observed in sleep quality at 18.50%, self-confidence at 18.31%, self-esteem at 17.86%, suicidal ideation at 15.00%, decision-making at 14.75%, and eating behavior at 14.44%. Smaller changes included hatred at 7.67% and communication at 4.36%. Hopelessness and relationship satisfaction carry negative percentages (-37.43% and -1.47%, respectively). These are artifacts of the per-client ratio rather than deterioration, and they arise from opposite ends of the scale: hopelessness from division by a very low baseline (3.00 falling to 2.30), and relationship satisfaction from a baseline already near the top of the normal band (38.30 rising to 38.50 on a 5-50 scale). In raw scale points, both improved, so neither represents true worsening. Ten of the 16 adolescent confidence intervals in Table 4 include zero, so most changes in this cohort are best described as indistinguishable from no change rather than as demonstrated improvement. The Stress Load Index improved to 21.91% +/- 13.41 (n = 9 complete cases).

Aggregate improvement and cross-population variability

Aggregating percent improvements across all 17 outcomes, mean improvement was 35.2% +/- 9.0 for adults, 47.3% +/- 9.2 for veterans, and 19.1% +/- 20.4 for adolescents. On the Emotional Checklist total, the designated primary symptom instrument, mean improvement pooled across all 50 clients was 46.63% (SD: 23.56). Relative to the instrument's normal band (per-item: 1-4; total: 12-48), the adult mean moved from above the band at baseline (70.85) to within it after treatment (38.80), and the veteran mean from 78.50 to 31.30, while the adolescent mean began within the band (42.50) and remained within it (28.90), so the pooled figure includes a cohort whose baseline mean did not indicate an elevated state. Veterans demonstrated both the highest mean improvement and the most consistent response, while adolescents showed the lowest and most variable.

Client-level response

Every client in the adult and veteran cohorts (40 of 40) showed directional improvement on the Emotional Checklist and the Behavior Control Checklist, with no instances of deterioration on either instrument. On the Relationship Satisfaction Scale, 17 of 20 adults and 18 of 20 veterans improved. In the adolescent cohort, 9 of 10 improved on the Emotional Checklist and the Behavior Control Checklist, while responses on the Relationship Satisfaction Scale were mixed.

Applying the Jacobson-Truax Reliable Change Index to instrument totals (Table 5), reliable improvement on the Emotional Checklist was achieved by 60.0% of adults, 90.0% of veterans, and 50.0% of adolescents. Reliable improvement on at least one of the three instruments was achieved by 95.0% of adults, 95.0% of veterans, and 50.0% of adolescents. The Relationship Satisfaction Scale showed the lowest reliable change rates in every cohort (35.0% of adults, 65.0% of veterans, and no adolescents), consistent with the smaller raw point changes on that instrument reported in Table 4.

**Table 5:** Reliable change (Jacobson-Truax) by instrument and cohort Standard error of the difference was computed from the cohort baseline SD and the instrument's standardized Cronbach's alpha. Threshold is 1.96 standard errors of the difference, the change in scale points required for classification as a reliable improvement. Computed from client-level raw data. In the client-level any-instrument rows, baseline SD, alpha, standard error, and threshold are not applicable, because those rows summarize whether a client met the criterion on any of the three instruments rather than on a single scale.

Instrument	Cohort	Baseline SD	Alpha	SE diff	Threshold	n reliable	n	% reliable
Emotional Checklist	Adults	23.60	0.91	10.01	19.62	12	20	60.0%
Emotional Checklist	Veterans	21.12	0.91	8.96	17.56	18	20	90.0%
Emotional Checklist	Adolescents	16.90	0.91	7.17	14.05	5	10	50.0%
Behavior Control Checklist	Adults	8.72	0.84	4.93	9.67	17	20	85.0%
Behavior Control Checklist	Veterans	8.46	0.84	4.79	9.38	17	20	85.0%
Behavior Control Checklist	Adolescents	12.38	0.84	7.00	13.73	3	10	30.0%
Relationship Satisfaction Scale	Adults	9.12	0.85	4.99	9.79	7	20	35.0%
Relationship Satisfaction Scale	Veterans	8.09	0.85	4.43	8.69	13	20	65.0%
Relationship Satisfaction Scale	Adolescents	9.25	0.85	5.07	9.93	0	10	0.0%
Any Instrument (Client Level)	Adults	n/a	n/a	n/a	n/a	19	20	95.0%
Any Instrument (Client Level)	Veterans	n/a	n/a	n/a	n/a	19	20	95.0%
Any Instrument (Client Level)	Adolescents	n/a	n/a	n/a	n/a	5	10	50.0%

Reliability

Reliability analysis showed strong internal consistency (Table 6), with a standardized Cronbach's alpha of 0.91 for the Emotional Checklist and 0.93 for the overall 22-item Emotional Fitness Checklist (both Excellent), and 0.84, 0.85, and 0.83 for the Behavior Control Checklist, Relationship Satisfaction Scale, and Stress Load Index composite (good), respectively. All coefficients meet the 0.80 threshold; classifications follow the George and Mallery convention.

**Table 6:** Reliability analysis (standardized Cronbach's alpha) Computed on the present study sample at baseline. Convention (George & Mallery): alpha >= 0.90 as excellent; 0.80-0.89 as good; 0.70-0.79 as acceptable. In the composite and overall coefficients, the Emotional Checklist is reverse-scored to align with valence. No coefficient is reported for the Gut Health Symptom Checklist, as no retained item-level data were available from these cohorts; it functioned as a referral gate rather than as a scored scale.

Assessment instrument	Standardized Cronbach's alpha	Reliability classification
Emotional Checklist	0.91	Excellent
Behavior Control Checklist	0.84	Good
Relationship Satisfaction Scale	0.85	Good
Stress Load Index (3-Instrument Composite)	0.83	Good
Emotional Fitness Checklist (22 Items)	0.93	Excellent

Effect sizes

Within-subject standardized mean change (d_z) for all 16 metrics across the three populations is presented in Table 7, computed from the raw client-level difference scores. Values range from 0.04 to 2.04. Because d_z is a within-subject statistic, these values are expected to exceed between-group Cohen's d benchmarks and are not interpreted against them.

**Table 7:** Within-subject standardized mean change (d_z) by population d_z = mean change / SD of change scores, computed from raw client-level difference scores. As a within-subject standardized mean change, d_z is typically larger than a between-group Cohen's d and should not be interpreted against between-group effect-size benchmarks. Adolescent love (emotional state) is n = 9.

Metric	Adults d_z	Veterans d_z	Adolescents d_z
1. Depression	1.60	1.80	1.22
2. Anxiety	1.49	2.04	1.70
3. Negative Self-Talk (Guilt)	1.89	1.46	0.81
4. Anger	0.70	1.92	1.15
5. Sleep Quality	0.89	1.62	0.56
6. Hopelessness	1.32	1.56	0.28
7. Self-Esteem	1.07	1.17	0.66
8. Eating Behavior	0.89	0.90	0.45
9. Decision-Making	0.93	1.25	0.35
10. Hatred (Self-Others)	0.86	1.14	0.19
11. Suicidal Ideation	0.81	1.05	0.62
12. Love (Emotional State)	1.31	1.29	1.37
13. Self-Confidence	1.45	1.25	0.63
14. Motivation and Focus	1.18	1.52	1.18
15. Communication	0.99	0.85	0.24
16. Relationship Satisfaction	0.96	1.25	0.04

Within-population paired t-test results are presented in Table 8. All 16 metrics reached p < 0.05 in the adult and veteran populations, and six of 16 reached p < 0.05 in the adolescent population.

**Table 8:** Within-population paired t-test results by metric (exact p-values) t = mean change / standard error of the change, computed on the raw client-level paired difference scores. df = n minus 1 (19 adults, 19 veterans, 9 adolescents; 8 for adolescent love (emotional state), where n = 9). p-values are two-tailed, uncorrected for multiple comparisons, and reported as exact values. Tests are descriptive rather than confirmatory.

Metric	Adults t	Adults p	Veterans t	Veterans p	Adolescents t	Adolescents p
1. Depression	7.16	8.39E-07	8.03	1.58E-07	3.84	4.00E-03
2. Anxiety	6.68	2.18E-06	9.10	2.34E-08	5.36	4.56E-04
3. Negative Self-Talk (Guilt)	8.43	7.60E-08	6.55	2.85E-06	2.58	3.00E-02
4. Anger	3.13	6.00E-03	8.60	5.61E-08	3.64	5.00E-03
5. Sleep Quality	3.96	8.40E-04	7.24	7.08E-07	1.77	1.11E-01
6. Hopelessness	5.90	1.10E-05	6.99	1.17E-06	0.89	3.98E-01
7. Self-Esteem	4.79	1.27E-04	5.23	4.75E-05	2.08	6.80E-02
8. Eating Behavior	3.97	8.26E-04	4.04	7.05E-04	1.41	1.91E-01
9. Decision-Making	4.16	5.27E-04	5.59	2.14E-05	1.11	2.97E-01
10. Hatred (Self-Others)	3.85	1.00E-03	5.11	6.17E-05	0.61	5.55E-01
11. Suicidal Ideation	3.64	2.00E-03	4.68	1.62E-04	1.96	8.10E-02
12. Love (Emotional State)	5.84	1.27E-05	5.78	1.43E-05	4.11	3.00E-03
13. Self-Confidence	6.47	3.37E-06	5.57	2.24E-05	1.99	7.70E-02
14. Motivation and Focus	5.30	4.09E-05	6.79	1.75E-06	3.74	5.00E-03
15. Communication	4.44	2.80E-04	3.82	1.00E-03	0.75	4.75E-01
16. Relationship Satisfaction	4.29	3.96E-04	5.59	2.15E-05	0.12	9.06E-01

Percent improvement

Percent improvements for all metrics, with the derived Stress Load Index, are presented in Table 9.

**Table 9:** Percent improvement by population, with the derived Stress Load Index Percent improvement is computed per client relative to the good-direction endpoint of each instrument: (before minus after) divided by before for the symptom-reduction items, and (after minus before) divided by after for the positive-direction items. The Stress Load Index is the mean of the three instrument percent improvements. Its standard deviation in percentage points is 14.78 for adults, 18.86 for veterans, and 13.41 for adolescents, and its 95% confidence interval is 29.57-43.41, 37.42-55.08, and 11.60-32.22, respectively. The adolescent Stress Load Index is a complete-case figure (n = 9). For adolescent hopelessness, the negative percentage is an artifact of dividing by a very low baseline; for adolescent relationship satisfaction, it arises from a baseline already near the top of the normal band. The raw point changes in Table 4 are positive in both cases.

Metric	Adults	Veterans	Adolescents	Flag
1. Depression	44.03%	57.87%	29.33%	
2. Anxiety	44.48%	54.47%	42.90%	
3. Negative Self-Talk (Guilt)	50.64%	51.37%	34.28%	
4. Anger	24.30%	63.95%	49.19%	
5. Sleep Quality	31.54%	55.79%	18.50%	
6. Hopelessness	43.69%	58.93%	-37.43%	Low-baseline artifact
7. Self-Esteem	40.82%	50.68%	17.86%	
8. Eating Behavior	35.61%	39.43%	14.44%	
9. Decision-Making	28.00%	43.72%	14.75%	
10. Hatred (Self-Others)	22.79%	44.32%	7.67%	
11. Suicidal Ideation	31.45%	46.29%	15.00%	
12. Love (Emotional State)	48.02%	47.68%	45.83%	
13. Self-Confidence	36.87%	35.93%	18.31%	
14. Motivation and Focus	31.51%	41.16%	29.37%	
15. Communication	27.94%	30.47%	4.36%	
16. Relationship Satisfaction	20.58%	34.99%	-1.47%	Near-ceiling artifact
17. Stress Load Index	36.49%	46.25%	21.91%	

## Discussion

This quality improvement initiative points to gains in both clinical outcomes and operational efficiency from standardizing measurement across a broad set of psychological metrics. A 67.5% reduction in treatment duration across all three populations, alongside improvements in measured outcomes, suggests that mechanism-focused protocols can be delivered in substantially less contact time while improvement is observed on the measured instruments. These findings map onto the three stated objectives: duration reduction (objective 1), measurable within-client improvement (objective 2), and maintained adherence, completion, and safety (objective 3).

The dual target and where the protocol acts

**Figure 1:**
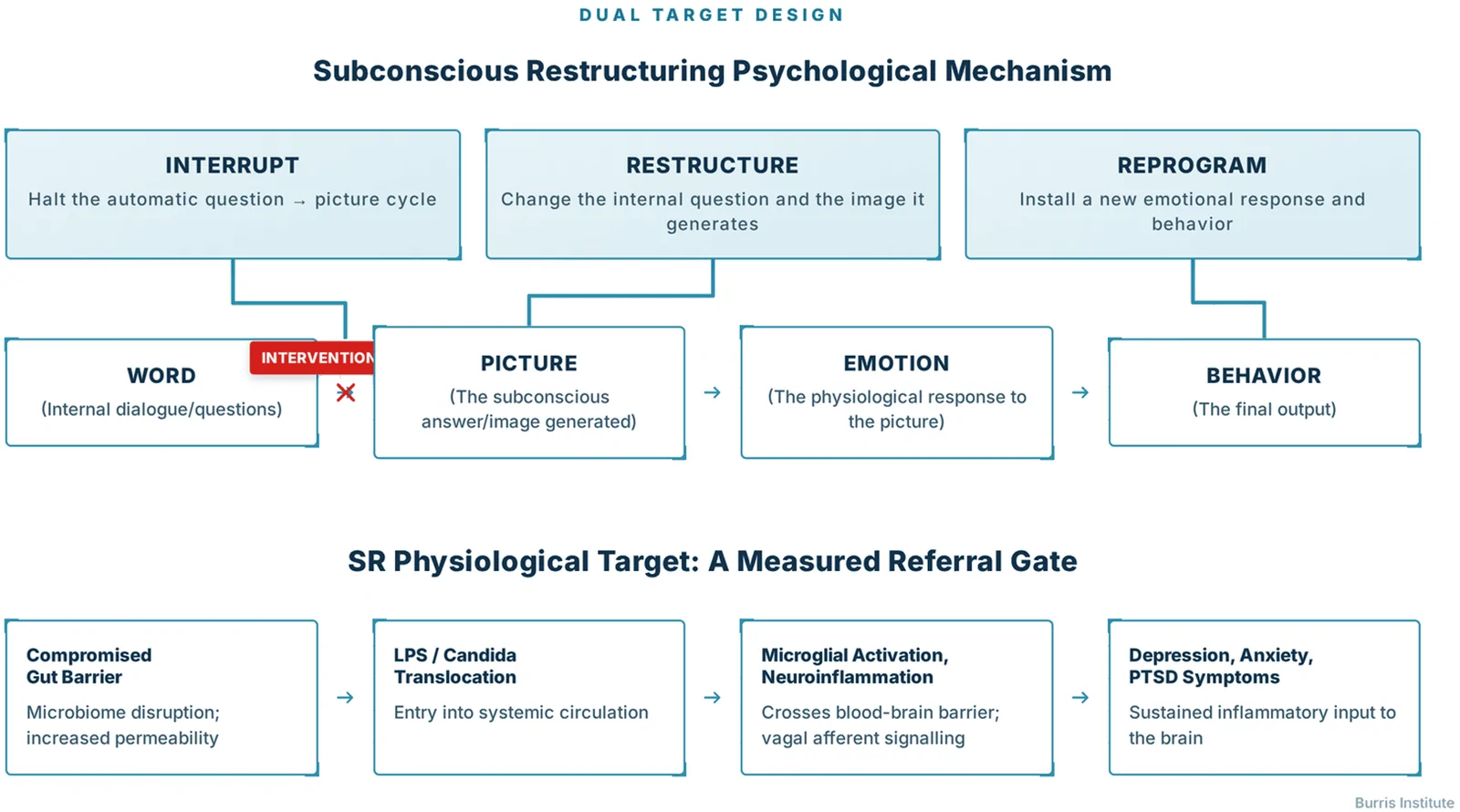
Subconscious Restructuring dual-target design Conceptual model of the two targets addressed by the Subconscious Restructuring (SR) protocol. The upper row, the SR psychological mechanism, shows the word-picture-emotion-behavior sequence and the three operations the protocol applies to it (interrupt, restructure, reprogram); the intervention marker denotes the word-to-picture transition at which the protocol acts. The lower row, the SR physiological target, shows the literature-derived inflammatory pathway that the measured referral gate screens for: a compromised gut barrier permits translocation of lipopolysaccharide and *Candida *into the systemic circulation, crossing the blood-brain barrier and activating microglia, with the resulting neuroinflammation implicated in depression, anxiety, and PTSD [14-26]. The Gut Health Symptom Checklist operationalizes this row as a referral gate under the Step 6 criterion. The pathway is the design rationale for the protocol’s dual target, not a hypothesis tested by this initiative.

Figure 1 sets out the two targets the protocol addresses and the point at which each is engaged. On the psychological row, the protocol treats the word-picture-emotion-behavior sequence as a chain rather than a set of independent symptoms, and it intervenes at the word-to-picture transition: Steps 2 and 3 restructure internal dialogue and the imagery it generates; Step 4 interrupts an established sequence at the identified cue; and Steps 5 and 7 consolidate the substitution through daily practice. Intervening early in the chain is what allows a fixed script to be applied across cohorts that differ in presenting concern, since the target is the mechanism generating the emotional state rather than the content of the state itself. On the physiological row, the published pathway runs from a compromised gut barrier through translocation of lipopolysaccharide and *Candida *into the systemic circulation, across the blood-brain barrier, to microglial activation and the neuroinflammation implicated in depression, anxiety, and PTSD [14-26]. The protocol does not act on that row. It screens it. Step 6 uses the Gut Health Symptom Checklist as a referral gate. The rationale for pairing the two rows is that psychological restructuring is unlikely to hold while an unaddressed inflammatory input persists, but that proposition was not tested here, and the physiological row of Figure 1 is therefore a design rationale rather than a finding. Establishing whether the referral gate performs as intended requires pairing the checklist with objective physiological markers, which is set out under Future Directions.

The reduction in treatment duration may translate into cost savings. Conventional treatment of 12-20 sessions is billed under the individual psychotherapy codes. Current Procedural Terminology code 90834 covers individual psychotherapy of 38-52 minutes, for which the 2026 Medicare national non-facility rate is $113.90 [42]. Code 90837 covers individual psychotherapy of 53 minutes or longer and corresponds to the full clock hour used in the duration comparison reported in the Results section; its 2026 Medicare national non-facility rate is $167.00 [42]. Both codes are reported here because they bracket ordinary outpatient practice and because the choice of code determines the size of the estimated saving. The national payment amount is used rather than a locality-adjusted rate because the practitioners delivering this protocol are distributed nationally. A cross-sectional analysis of 175,083 psychotherapy providers listed in a national directory reported a mean cash-pay session rate of $143.26 against a mean Medicaid fee-for-service rate of $82.77 [43]. These two figures are per-session averages that are not stratified by session length and therefore cannot be assigned to either code.

Expressed in equivalent billable units, the weighted mean SR intervention time of 5.2 hours corresponds to 6.9 units of 45-minute contact. Valued at the 90834 rate, a conventional course of 16 sessions costs approximately $1,822 per patient, compared with approximately $790 for the SR protocol, a saving of approximately $1,033. Against the 12-session and 20-session bounds, the saving ranges from approximately $577 to $1,488. At the cash-pay rate, the corresponding savings for a 16-session course are approximately $1,299, and at the Medicaid rate, approximately $750. Valued at the 90837 rate, the weighted mean SR intervention time of 5.2 hours corresponds to 5.2 units of 60-minute contact, compared with a conventional 16 units. A conventional 16-session course then costs approximately $2,672 per patient, compared with approximately $868 for the SR protocol, a saving of approximately $1,804. Against the 12- and 20-session bounds, the savings range from approximately $1,136 to $2,472. The proportional reduction on this basis is 67.5% rather than 56.7%.

Because the comparison is proportional to contact time, the percentage reduction depends on the assumed billable unit: 56.7% when the conventional course is expressed in 45-minute units under 90834, and 67.5% when it is expressed in 60-minute units under 90837. These figures are projections derived from published reimbursement rates and reported session fees, not costs measured in this study, and they exclude practitioner training, platform, and administrative overhead. For healthcare systems, the reduction in total contact time could translate to serving more patients with existing resources.

The standardized, protocol-driven approach offers promise for global scalability, especially in low- and middle-income countries where specialist shortages create service gaps [44]. The scripted training model enables rapid workforce development, while cloud-based infrastructure supports quality assurance and outcome monitoring. Placement within existing services is a separate implementation question. A scoping review of mental health integrated care models in primary care identifies the factors that contribute to effective implementation, and those factors bear directly on any attempt to situate a protocol of this kind inside routine care rather than alongside it [45].

Comparison with published outcomes

No controlled comparison against an active alternative was undertaken, and SR has not been evaluated head-to-head against any established modality, so the following places the metrics reported here alongside published benchmarks measured in the same way rather than against any specific intervention. Because the three cohorts differ in age, setting, and presenting concern, each is compared against the literature appropriate to it rather than against a single common comparator.

Regarding treatment duration, the systematic review of routinely delivered psychological therapies reported optimal doses ranging from approximately 4 to 26 sessions, depending on the setting, population, and outcome measure [4]. The weighted mean of 5.2 contact hours reported here sits at the low end of that span, which is the sense in which the reduction represents a departure from routine dosing rather than a demonstration of superior efficacy. On client-level response, the most substantial published benchmark is the English Improving Access to Psychological Therapies program, which obtains paired before-and-after scores for the large majority of patients treated and publishes reliable improvement rates nationally; national reporting has placed reliable improvement in the region of 60%-67% of patients completing a course of treatment [46]. Reliable improvement on the primary symptom instrument in the present initiative was 60.0% of adults and 90.0% of veterans, which falls at the lower edge of that band and above it, respectively. The comparison is bounded in an important way: the instrument used here is an original measure without a validated diagnostic threshold, no criterion measure was administered, and the reliable change criterion was computed from this sample's own baseline standard deviation and alpha rather than from published norms, so these rates are not interchangeable with program-level figures derived from validated scales.

Evidence from the population most similar to the veteran cohort here bears on the same question. In a study of psychotherapy for depression delivered across Veterans Affairs primary care and specialty mental health clinics, the good-enough level model fit better than the dose-effect model in both settings; veterans receiving fewer sessions improved faster than those receiving more; veterans receiving four to eight sessions in primary care achieved comparable levels of symptom response, and those receiving 20 sessions showed minimal response [47]. That finding does not validate the protocol reported here, and the outcome measure and setting differ. It does establish that within a large veteran population, additional contact time beyond a point has not been associated with additional improvement, which is the assumption that the duration comparison in this report depends upon.

On delivery format, compressed scheduling has been examined directly in the population most similar to the veteran cohort here. A non-inferiority randomized controlled trial in Australian military personnel and veterans compared massed with standard prolonged exposure and found the compressed format non-inferior at 12-month follow-up [48]. That finding is relevant to the pattern observed in this initiative, where the veteran cohort received the shortest protocol yet recorded the largest and most consistent gains, and it supports the view that concentrating contact time is not inherently a compromise. For adolescents, the appropriate comparator is the single-session literature. A meta-analysis of 50 randomized controlled trials in 10,508 youths reported a mean postintervention effect of Hedges g equal to 0.32 for single-session interventions, with smaller effects in older adolescents than in younger children [49]. The smaller and more variable adolescent response reported here is therefore consistent with what that literature would predict of a one-contact format delivered to a cohort with a mean age of 15.9 years, and supports reading the adolescent result as a function of the delivery structure rather than as a ceiling on what the protocol can achieve in that age group.

Applicability by presenting concern and age

The design does not support claims about which diagnoses the protocol suits. Clients were not assigned diagnostic labels, consistent with the mechanistic rather than symptomatic operationalization described in the Materials and Methods section, and no structured diagnostic interview was administered, so the cohorts cannot be characterized in diagnostic terms. What can be stated is the range of presenting circumstances actually represented. The veteran cohort was in treatment for service-related trauma, traumatic brain injury, and behavioral health conditions, including PTSD, depression, and anxiety, and recorded the largest gains. The adult civilian cohort enrolled without eligibility restriction and presented with a mean Emotional Checklist total above the instrument's normal band, indicating elevated symptom burden at entry, and improved consistently across every metric. The adolescent cohort entered with a mean total already inside the normal band, which limits what improvement on that instrument can demonstrate in that group. Across the three cohorts, the age range spanned 13 to the mid-60s, and improvement in raw scale points was observed in every metric at every age band, though with markedly reduced precision in the youngest. On the available evidence, the protocol is best described as having been delivered to adults and adolescents with self-reported emotional symptom burden of varying severity, with the strongest signal in a trauma-exposed adult population and the weakest in adolescents receiving a single session. Whether it is suited to any specific diagnosis, and whether the gut health referral gate contributes anything to outcome in any of them, are questions for the controlled work described below.

Limitations

This QI evaluation has several limitations. The sample of 50 clients limits statistical power. The absence of comparison groups prevents assessment relative to alternatives. Reliance on self-report may introduce bias. The absence of long-term follow-up prevents assessment of durability. The three cohorts were not contemporaneous (2011, 2016, 2018), and because cohort, practitioner, protocol, and collection year varied together, between-population differences cannot be attributed to any single factor. The adolescent cohort was a convenience sample and received a non-optimal single-session structure. Adolescence has additionally been proposed as a distinct and under-examined developmental window in microbiome-gut-brain axis communication [50], so the smaller and more variable response observed in this cohort should not be read as a ceiling on what an optimized adolescent protocol might achieve. Depressive symptom burden is operationalized as the Emotional Checklist total rather than measured with a validated diagnostic instrument; no external criterion measure was administered, and the adolescent cohort's baseline mean fell within the instrument's normal band, so the pooled improvement figure should be read as change on this instrument rather than as change in diagnosed depression. Fifteen of the 48 change-score distributions departed from normality; a Wilcoxon signed-rank sensitivity analysis agreed with the parametric results in all 48 comparisons, so this did not alter the reported conclusions.

The duration reduction is a ratio against a published benchmark rather than a measured treatment effect, and its magnitude depends on which benchmark bound is assumed; it is therefore reported as a range of approximately 56.7%-74.0% against the 12-hour and 20-hour bounds, with 67.5% as the point estimate against the 16-hour midpoint. Expressed instead in 45-minute billable units under CPT code 90834, the same comparison against a 16-session course yields 56.7%. These two 56.7% figures are numerically identical by coincidence and are not the same quantity: the first is the reduction against the 12-hour bound on a clock-hour basis, and the second is the reduction against a 16-session course expressed in 45-minute units. Practitioner documentation time was not measured on either side of the comparison. The benchmark itself is also a description of typical delivered treatment length rather than a demonstrated necessary dose, so the reduction reported here measures a departure from customary practice and should be read on that basis.

As an observational, single-arm evaluation, the design cannot separate the effects of the protocol from selection bias arising from convenience sampling, expectancy effects associated with a brief and intensive format, or practitioner effects, since a single practitioner delivered each cohort. Fidelity to the protocol was not independently verified: no adherence rating, session audit, or clinical supervision was conducted, and protocol consistency rested on the scripted guidelines and platform-captured session data rather than on observed delivery, with the adolescent cohort recorded on paper rather than in the platform. One client scored above the suicidal-ideation referral threshold following intervention and declined the referral offered, which illustrates that the safety pathway depends on client acceptance. The comparisons drawn above against published benchmarks are indirect: they place figures obtained here alongside figures obtained elsewhere on different samples with different instruments, and they cannot substitute for a controlled comparison. Finally, the Gut Health Symptom Checklist is a specified component of the protocol and was completed by the client at every session, but structured retention of those responses in the platform did not begin until 2021, so no gut health data from these cohorts were available for analysis. The limitation is one of data retention rather than of protocol delivery. 

Future directions

Priority areas include comparative effectiveness evaluations, longer-term outcome tracking, systematic collection and analysis of gut health data alongside psychometric outcomes, and the development of population-specific protocols (particularly an optimized adolescent protocol). Future software development priorities include automated risk flagging for mandatory referrals, script-guided protocol delivery, intelligent progress tracking, and, pending client approval, integration with broader medical databases for a large-scale study. Pairing the Gut Health Symptom Checklist with objective physiological markers would allow the proposed relationship between gut and emotional status to be tested directly and would establish whether the referral gate performs as intended.

## Conclusions

This QI initiative suggests that implementing a standardized brief emotional fitness protocol delivered through the SR framework may be associated with improvements in both clinical outcomes and service delivery efficiency. Consistent with the three stated objectives, the systematic approach reduced treatment duration by approximately 67.5% against the midpoint of the conventional benchmark, within a range of approximately 56.7%-74.0% against the 12-hour and 20-hour bounds of that benchmark, produced measurable within-client improvement across the measured outcomes in all three populations, and maintained 100% adherence, completion of the psychometric assessments, and safety with high internal consistency across instruments; gains were the largest in veterans and smallest and most variable in adolescents.

As a single-arm evaluation without a comparison group, these findings are hypothesis-generating; controlled studies are needed to confirm effects on mental health outcomes. The behavioral microbiome model informed the design of the protocol but was not evaluated here; testing whether integrated gut barrier assessment contributes to outcome is the next step.

## Figures and Tables

**Figure 2:**
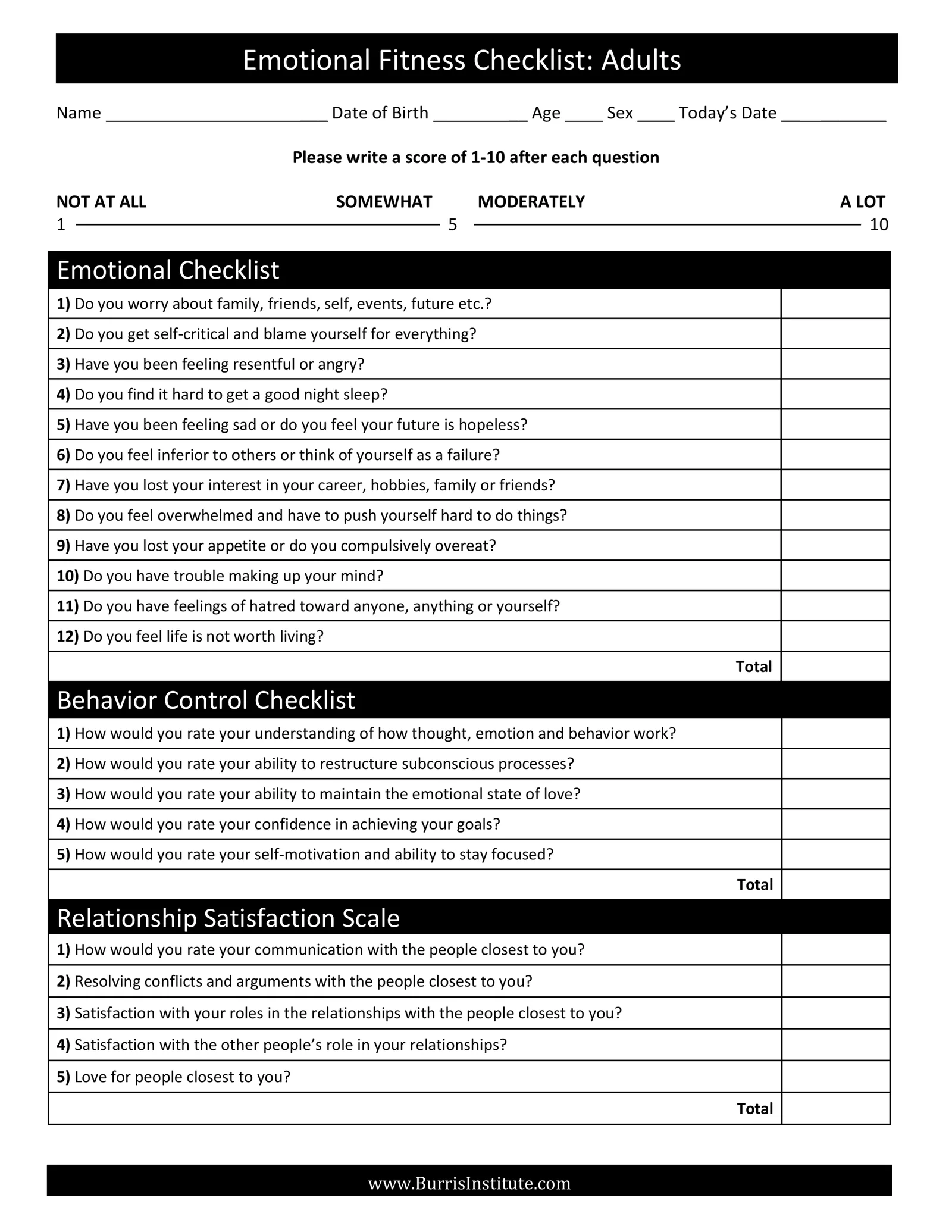
Emotional Fitness Checklist: adults This 22-item instrument comprises the Emotional Checklist (12 items), the Behavior Control Checklist (five items), and the Relationship Satisfaction Scale (five items). All items are scored on a 1-10 Likert metric anchored at 1 (not at all) and 10 (a lot), with a total recorded for each instrument. The adult civilian and veteran cohorts completed the instrument on the Burris Connect platform and the adolescent cohort on paper; item content and scoring are identical in both modes and are unchanged from the versions administered in 2011, 2016, and 2018. The instrument is available at no cost and is copyrighted by the Burris Institute. Reproduced with permission of the copyright holder.

**Figure 3:**
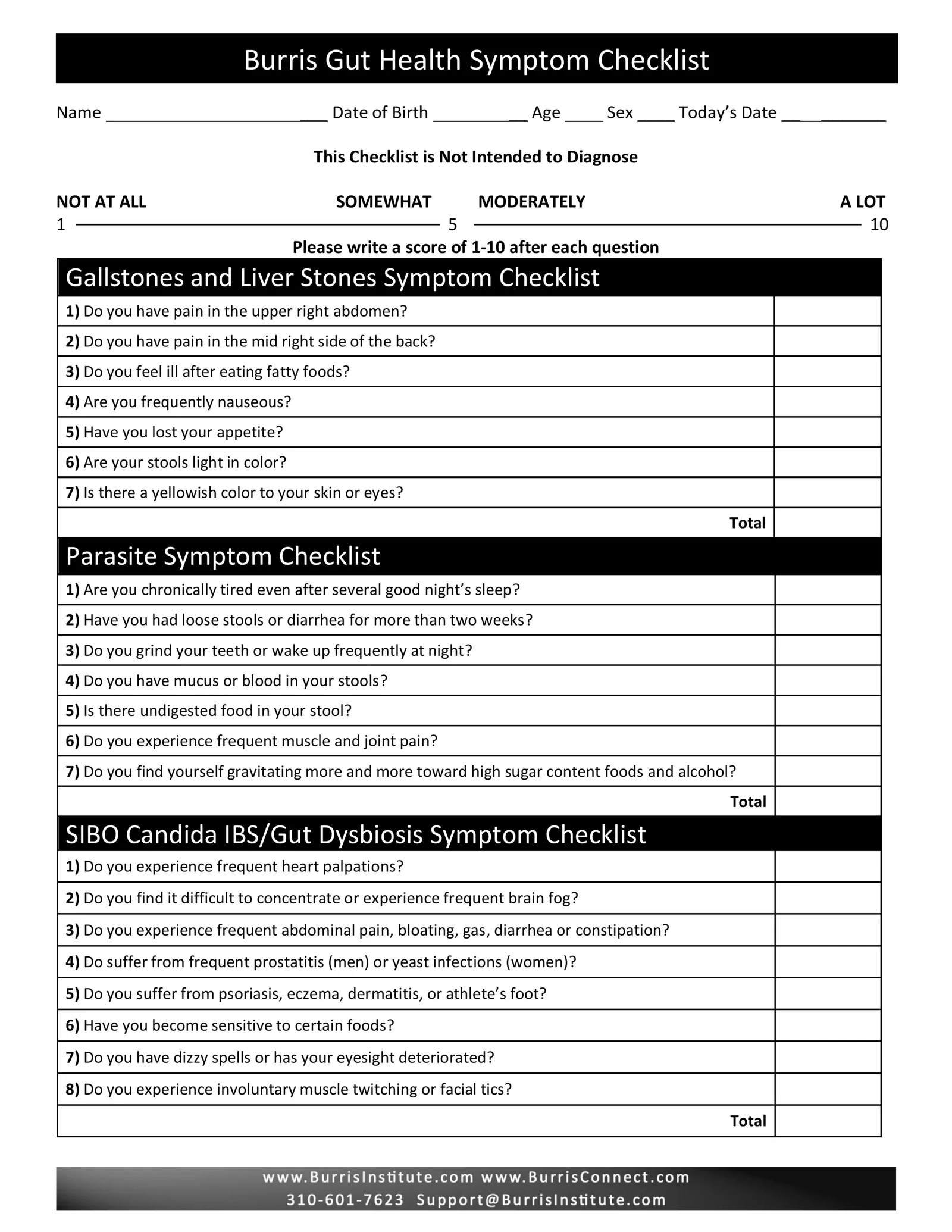
Burris Gut Health Symptom Checklist This 22-item referral-gate instrument comprises gallstones and liver stones (seven items), parasites (seven items), and SIBO, Candida, IBS, and gut dysbiosis (eight items), each scored on the same 1-10 Likert metric with a section total. The instrument is non-diagnostic. As currently distributed, it is presented with a disclaimer that the respondent must acknowledge before the instrument can be completed, stating the non-diagnostic limitation and directing the respondent to a qualified health care provider. Administration of this checklist was not systematically documented in the platform until 2021, and no gut-health data from it were available for the cohorts reported here. The instrument is available at no cost and is copyrighted by the Burris Institute. Reproduced with permission of the copyright holder.
